# Paradoxical Potentiation of Acid-Sensing Ion Channel 3 (ASIC3) by Amiloride *via* Multiple Mechanisms and Sites Within the Channel

**DOI:** 10.3389/fphys.2021.750696

**Published:** 2021-10-15

**Authors:** Daniel S. Matasic, Nicholas Holland, Mamta Gautam, David D. Gibbons, Nobuyoshi Kusama, Anne M. S. Harding, Viral S. Shah, Peter M. Snyder, Christopher J. Benson

**Affiliations:** ^1^Division of Cardiovascular Medicine, Department of Internal Medicine, University of Iowa, Iowa City, IA, United States; ^2^Department of Molecular Physiology and Biophysics, University of Iowa, Iowa City, IA, United States; ^3^Department of Neuroscience and Pharmacology, University of Iowa, Iowa City, IA, United States; ^4^Iowa City VA Healthcare System, Iowa City, IA, United States

**Keywords:** acid-sensing ion channels, channel gating, proton sensing, amiloride, whole-cell patch clamp

## Abstract

Acid-Sensing Ion Channels (ASICs) are proton-gated sodium-selective cation channels that have emerged as metabolic and pain sensors in peripheral sensory neurons and contribute to neurotransmission in the CNS. These channels and their related degenerin/epithelial sodium channel (DEG/ENaC) family are often characterized by their sensitivity to amiloride inhibition. However, amiloride can also cause paradoxical potentiation of ASIC currents under certain conditions. Here we characterized and investigated the determinants of paradoxical potentiation by amiloride on ASIC3 channels. While inhibiting currents induced by acidic pH, amiloride potentiated sustained currents at neutral pH activation. These effects were accompanied by alterations in gating properties including (1) an alkaline shift of pH-dependent activation, (2) inhibition of pH-dependent steady-state desensitization (SSD), (3) prolongation of desensitization kinetics, and (4) speeding of recovery from desensitization. Interestingly, extracellular Ca^2+^ was required for paradoxical potentiation and it diminishes the amiloride-induced inhibition of SSD. Site-directed mutagenesis within the extracellular non-proton ligand-sensing domain (E79A, E423A) demonstrated that these residues were critical in mediating the amiloride-induced inhibition of SSD. However, disruption of the purported amiloride binding site (G445C) within the channel pore blunted both the inhibition and potentiation of amiloride. Together, our results suggest that the myriad of modulatory and blocking effects of amiloride are the result of a complex competitive interaction between amiloride, Ca^2+^, and protons at probably more than one site in the channel.

## Introduction

Acid-sensing Ion Channels (ASICs) are H^+^-gated cation channels within the degenerin/epithelial sodium channel (DEG/ENaC) family (Waldmann et al., 1997b). Primarily expressed in neurons of the central and peripheral nervous systems, ASICs have emerged as important mediators in a variety of physiological processes including pain sensation (Sluka et al., 2009), learning behaviors (Wemmie et al., 2002), and fear conditioning (Wemmie et al., 2003, 2004). In general, ASICs exhibit a rapid inward sodium current when activated by extracellular protons that subsequently desensitizes over the course of milliseconds to seconds in the continued presence of protons. Of the six homologous subunits identified (*ASIC*1a, -1b, -2a, -2b, -3, -4), ASIC3 is the key component in the peripheral nervous system, governing the excitability of a variety of sensory neurons (Waldmann et al., 1997a; Benson et al., 2002; Hattori et al., 2009; Khataei et al., 2020). In addition to its expression profile, ASIC3-containing channels are unique by generating sustained currents from incomplete desensitization of channels in the continued presence of protons. This sustained current occurs around pH 7 via an overlap between pH-dependent activation and desensitization dose-response curves to generate a “window” current (Yagi et al., 2006).

Amiloride is the established and historical non-selective inhibitor of ASIC channels and thought to block current by physical occlusion at a binding site within the pore. Previous studies established that amiloride block was dependent on the potential difference across the membrane (Adams et al., 1999). As a weak base (pKa = 8.7), the cationic amiloride is thought to gravitate toward the electrical field within the pore, resulting in the block of Na^+^ current (Kellenberger and Schild, 2002). At more positive membrane potentials, the positively charged amiloride is driven out of the pore and amiloride-mediated inhibition is diminished. However, in addition to blocking ASIC currents, amiloride was found to paradoxically potentiate sustained currents in both ASIC3-containing channels and “Deg”-mutant ASIC2a-containing channels (Adams et al., 1999; Yagi et al., 2006). Interestingly, this potentiation by amiloride was not influenced by the transmembrane potential difference and therefore amiloride was speculated to be acting at a different site, likely on the extracellular face (Adams et al., 1999). Insights into the potential structural binding sites of amiloride were advanced with the crystallization of the chicken ASIC1 trimeric complex which demonstrated complexity of extracellular subdomains and labeled akin to its “hand” appearance: palm, thumb, finger, β-ball, and knuckle (Jasti et al., 2007; Gonzales et al., 2009). However, the structural-functional relationship of the paradoxical potentiation of amiloride on ASIC3 remains unclear.

Investigation into the underlying mechanisms of amiloride-induced potentiation has been aided by the discovery of synthetic compounds such as 2-guanidine-4-methylquinazoline (GMQ) that have emerged as activators of ASIC3 at neutral pH (Yu et al., 2010; Li et al., 2011). GMQ activates ASIC3 sustained currents by inducing an acidic shift in the pH-dependence of inactivation while provoking an alkaline shift in the pH-dependence of activation (Alijevic and Kellenberger, 2012). These effects, like amiloride-induced potentiation, increase overlap between the pH-dependent curves and thereby generate a larger sustained window current. It is evident amiloride-induced activation shares similarities to the complexities seen with GMQ-induced activation.

Characterization of GMQ on ASIC3 led to the identification of a non-proton ligand-sensing domain involving residues within the lower “palm” extracellular subdomain, E79 and E423, that were critical for amiloride-induced and GMQ-induced sustained currents (Yu et al., 2010; Li et al., 2011). However, subsequent studies found that disruption of these residues did not completely abolish the effect of GMQ to alter pH-dependent gating of ASICs (Alijevic and Kellenberger, 2012). Together, these studies suggest that the purported non-proton ligand-sensing domain may not be the sole site underlying these modulatory effects. As further evidence of a more complex mechanism, studies involving chimeras of ASIC1a and ASIC3 demonstrated involvement of both extracellular and transmembrane/cytosolic domains in amiloride-induced activation of ASIC3 (Besson et al., 2017). Here in these studies, we further characterize the paradoxical potentiation of ASIC3 by amiloride and investigate its determinants.

## Materials and Methods

### DNA Constructs

Rat ASIC3 in pMT3 vectors were cloned as previously described (Eshcol et al., 2008). Point mutations were generated by site-directed mutagenesis using the QuikChange kit (Stratagene, La Jolla, CA) and sequenced at the Iowa Institute of Human Genomics facility at the University of Iowa. Green fluorescent protein (GFP; pGreen Lantern) was obtained from Life Technologies (Thermo Fisher Scientific, Carlsbad, CA).

### Cell Culture and Transfection

Chinese Hamster Ovarian (CHO-K1) cells (ATCC, Manassas, Virginia) were cultured in F12 medium (GIBCO, Carlsbad, CA) containing 10% fetal bovine serum (FBS) and 1% penicillin-streptomycin. Cells were cultured at 37°C, 5% CO_2_, and plated at approximately 10% confluency into 35 mm dishes for electrophysiological studies. Cells were transfected using the lipid transfection reagent Transfast (Promega, Madison, WI) following manufacturer’s instructions. Wildtype rASIC3 cDNA (0.18 μg/1.5 ml) was transfected with rASIC3 G445C (0.162 μg/1.5 ml) at a 1:10 ratio. All cells were transfected with GFP (0.330 μg/1.5 ml) to assist with identification of transfected cells.

### Whole-Cell Patch Clamp Electrophysiology

Whole-cell patch clamp (−70 mV) recordings in CHO-K1 cells were performed at room temperature with an Axopatch 200B amplifier (Axon Instruments, Foster City, CA). Traces were acquired and analyzed with PatchMaster/FitMaster (HEKA Electronics, Lambrecht, Germany) or pClamp8 (Molecular Devices, San Jose, CA) and Prism (GraphPad Software, La Jolla, CA). Capacitive currents were compensated for and recorded for normalization of peak current amplitudes. Glass electrophysiology pipettes (2–4 MΩ) for most experiments contained 100 KCl, 10 EGTA, 40 HEPES, and 5 MgCl_2_ (mM), pH 7.4 with KOH. For experiments involving positive voltages, NaCl was used in place of KCl. External solutions typically contained 120 NaCl, 5 KCl, 1 MgCl_2_, 2 CaCl_2_, 10 HEPES, and 10 MES (mM), pH adjusted to target by TMA-OH and osmolarity was balanced with TMA-Cl. Ca^2+^ levels in external solutions were modified for some experiments as noted in results. Nominal divalent solutions contained no added Ca^2+^ or Mg^2+^. Rapid extracellular solution exchanges were made using a computer-driven solenoid valve system. Kinetics of desensitization were fit with single exponential equations, and time constants (τ) reported. pH-dependent activation and steady-state desensitization (SSD) curves were fit to the Hill equation using GraphPad Prism. Data are means ± SEM. Statistical differences were assessed as described in figure legends using GraphPad Prism.

## Results

### Inhibition and Potentiation of Acid-Sensing Ion Channel 3 Currents by Amiloride

**FIGURE 1:**
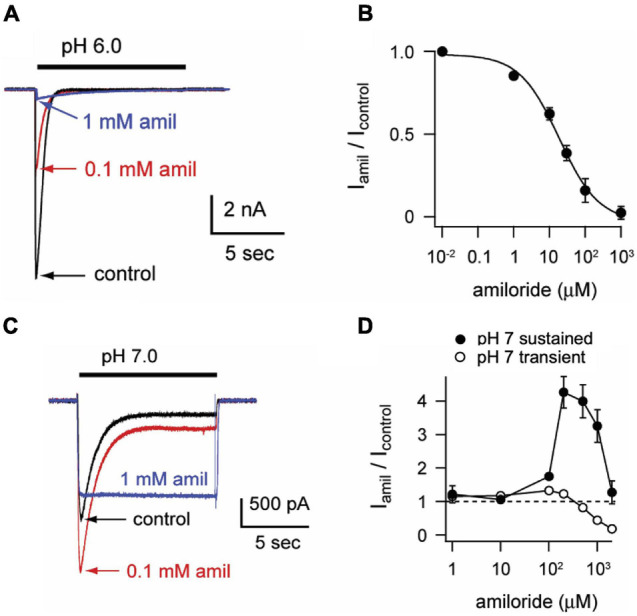
Amiloride paradoxically inhibits or potentiates ASIC3. **(A)** Overlay of representative currents recorded from CHO cells transfected with ASIC3 evoked by stepping from pH 8 (containing no amiloride) to pH 6 in the absence (control) or presence of 100 μM or 1 mM amiloride. **(B)** Dose-response curve for amiloride inhibition of pH 6-evoked currents. Line is fit of Hill equation (IC_50_ = 18.6 μM; *n* = 5–8 for each data point). **(C)** Overlay of pH-7 evoked currents in absence or presence of indicated amiloride concentrations. **(D)** Dose-responses of amiloride on the peak (transient) and sustained (measured at the end of 10 s pH 7 application) components of pH 7-evoked currents (*n* = 8–11 for each data point). Note that data points above the dashed line represent amiloride potentiation and those below represent inhibition.

**FIGURE 2:**
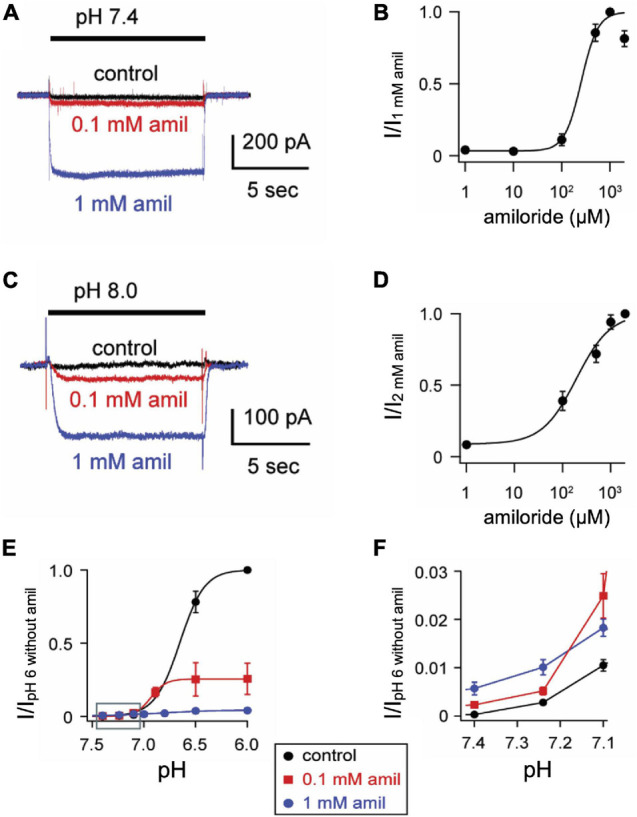
Amiloride increases the pH sensitivity of activation of ASIC3. **(A)** Representative superimposed ASIC3 currents evoked from a control solution of pH 8.0 (no amiloride) to pH 7.4 (containing the indicated amiloride concentrations at 0, 0.1 mM, and 1 mM). **(B)** Dose-response curve for amiloride on pH 7.4-evoked currents (*n* = 7–11 for each data point). Line is fit of Hill equation (EC_50_ = 255 μM). **(C)** Representative superimposed currents from a control solution of pH 8.0 (no amiloride) to pH 8.0 (containing the indicated amiloride concentrations). **(D)** Dose-response curve for amiloride on pH 8.0-evoked currents (*n* = 8). Line is fit of Hill equation (EC_50_ = 193 μM). **(E)** pH dose-response curves for activation in 0 mM (control), 0.1, and 1 mM amiloride. Data are acquired by stepping from pH 8.0 (with 0 mM amiloride) to the indicated test solutions (containing the indicated amiloride concentrations) and are normalized to the peak currents evoked by pH 6.0 in no amiloride (*n* = 4–9 for each data point). Lines are fits of Hill equations (pH_50_ = 6.7, 6.9, and 6.8 for 0, 0.1, and 1 mM amiloride, respectively). Note the error bars are often smaller than the markers. **(F)** A graph of the boxed area in **(E)** with expanded axes demonstrating the potentiating effect of amiloride at the foot of the pH dose-response curves (one-way ANOVAs at pH 7.40 and 7.24 found differences between groups (*p* < 0.01), with Dunnit’s multiple comparisons showing *p* < 0.01 for amiloride 1 mM vs. control at pH 7.40 and 7.24).

To characterize the paradoxical effects of amiloride, we studied pH-evoked currents from ASIC3 channels expressed in CHO cells. At a maximally activating pH 6, amiloride caused a classic dose-dependent block (IC_50_ = 18.6 μM, Figures 1A,B). However, at a more modest pH 7 activation, a dose-dependent paradoxical stimulatory effect was observed (Figure 1C). The pH 7-evoked currents demonstrate two components: a transient component that undergoes desensitization (like the pH 6-evoked currents) and a sustained component due to an overlap of the activation and SSD pH-dependent dose-response curves in the pH 7 range (Yagi et al., 2006). Paradoxical potentiation of both the transient (*p* < 0.02 by paired student’s *t*-test) and sustained (*p* < 0.02 by paired student’s *t*-test) pH 7-evoked currents is seen with 0.1 mM amiloride. However, at higher doses of amiloride (1 mM), the transient component is blocked but the sustained component is potentiated. Interestingly, amiloride increasingly potentiated the pH 7.0-evoked sustained current up to a concentration of 200 μM, but current was diminished at higher doses of amiloride (Figure 1D). Even at neutral pH (7.4) and more alkaline pH (8.0), amiloride stimulates a sustained current in a dose-dependent manner (Figures 2A–D). However, at pH 9.0 amiloride was unable to evoke current (data not shown), implying that amiloride potentiates pH-evoked currents, rather than functioning as a channel activator on its own. Note that the doses of amiloride required for potentiation (Figures 1D, 2B,D) all indicate an IC_50_ of greater than 100 μM, which is higher than the dose of amiloride required to block current (IC_50_ = 18.6 μM, Figure 1B). pH activation curves in the absence and presence of amiloride further demonstrate these paradoxical effects (Figure 2E); at more acidic pH values that maximally activate ASIC3, amiloride blocks current. However, at the foot of the activation curve, there is a shift to the left such that pH-evoked currents are potentiated (Figure 2F). In summary, as previously described (Yagi et al., 2006; Li et al., 2011), amiloride either blocks or potentiates ASIC3 current dependent on both the activating pH and the dose of amiloride.

### Amiloride Alters pH-Dependent Steady-State Desensitization

**FIGURE 3:**
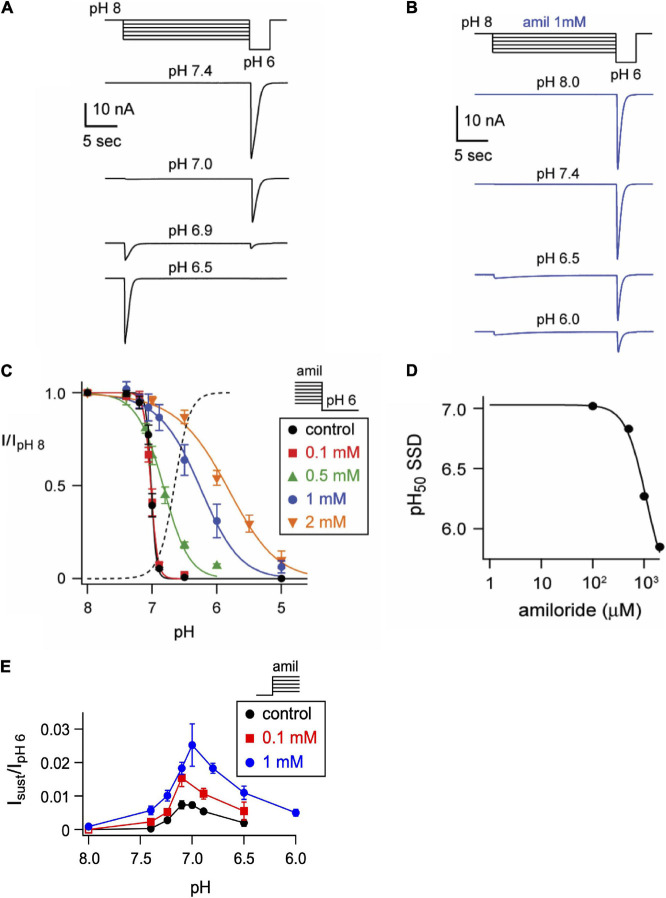
Amiloride inhibits the pH-dependent steady-state desensitization of ASIC3. **(A)** Representative ASIC3 currents evoked by stepping from pH 8 to the indicated desensitizing pH application for 20 s, following by a pH 6 test application. **(B)** Currents were recorded as in *A*, except the desensitizing 20 s pH solutions contained 1 mM amiloride. **(C)** pH dose-response curves for steady-state desensitization (SSD) recorded as in *A*, in control (no amiloride) and the indicated amiloride concentration solutions normalized to the currents evoked by stepping from pH 8 to pH 6 (*n* = 3–10 per data point). Lines are fits of the Hill equation (pH_50_ = 7.0, 7.0, 6.8, 6.3, and 5.9 for 0, 0.1, 0.5, 1, and 2 mM amiloride, respectively). Dashed line is fit of Hill equation for dose-response of pH activation in absence of amiloride from Figure 2E. **(D)** A plot of the amiloride dose-response on the pH_50_ values of steady-state desensitization measured from data in *C*. Line is fit of Hill equation (EC_50_ = 1.1 mM). **(E)** pH dose-response for activation of sustained currents (measured at the end of 10 s pH application) in 0 mM (control), 0.1, and 1 mM amiloride. Data are acquired by stepping from pH 8.0 (with 0 mM amiloride) to the indicated test solutions (containing the indicated amiloride concentrations) and are normalized to the peak currents (transient) evoked by pH 6.0 in no amiloride (*n* = 4–10 per data point).

One mechanism by which amiloride potentiates ASIC acid-evoked currents is by inhibiting pH-dependent SSD (Besson et al., 2017). To further explore the effect of amiloride on SSD, cells were conditioned with extracellular solutions at desensitizing pH values (8.0–5.0) for 20 s, followed by a pH 6 test application (Figures 3A,B). When added to the conditioning solution, SSD was shifted to the right by amiloride at doses greater than 0.1 mM (Figure 2C) in a dose-dependent manner (pH_50_ values of SSD in varying doses of amiloride show a IC_50_ ≥ 1.1 mM amiloride; Figure 3D).

ASIC3 is unique among ASIC channels in that there exists an overlap between the pH range of activation and SSD, thus creating a sustained “window current” whereby channels are activated, but incompletely desensitized. Consistent with the shift to the left at the foot of the activation curves (Figure 2F), and the shift to right of the SSD curves (Figure 3C), we found that amiloride generated larger sustained currents in this pH range (Figure 3E).

### Amiloride Slows the Rate of Desensitization and Facilitates Recovery From Desensitization

**FIGURE 4:**
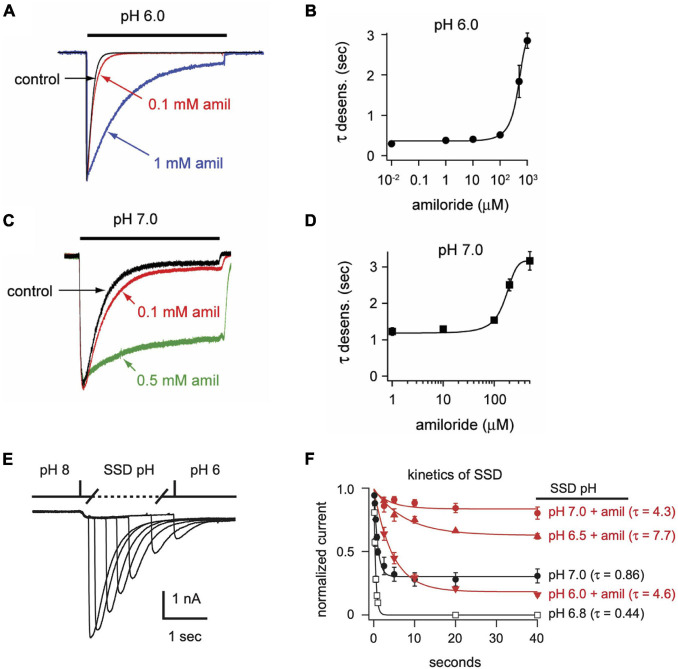
Amiloride slows the rate of desensitization of ASIC3. Overlays of **(A)** pH 6- or **(C)** pH 7-evoked normalized currents in the absence (control) or presence of amiloride at the indicated concentrations. Acidic pH was applied for 10 s. Amplitudes of pH 6-evoked currents (nA): control 18.6, 0.1 mM 5.66, 1 mM 0.70; pH 7-evoked currents (nA): control 1.05, 0.1 mM 1.40, 0.5 mM 0.78. **(B,D)** Are the mean time constants of desensitization (τ) as measured from single exponential fits to the falling phase of pH 6- **(B)** and pH 7-evoked currents **(D)** in varying concentrations of amiloride (*n* = 4–8 per data point). Lines are sigmoid fits. **(E)** An overlay of currents evoked by stepping from pH 8 to pH 7 solution for variable time periods (see X axis of *F*), following by a pH 6 test application. This protocol measures the time to steady-state desensitization (SSD). **(F)** Mean data of the time to SSD as collected in *E* for currents that underwent SSD in the indicated pH solutions labeled at the right of each curve (*n* = 3–10 per data point). Solutions with amiloride (red) contained 1 mM amiloride. Lines are fits of single exponentials of the means and time constants (τ) are shown.

In addition to differentially influencing current amplitudes, amiloride dose-dependently prolongs the desensitization of ASIC3 channels. Overlaying of pH 6-evoked currents with normalized amplitudes (Figure 4A) demonstrates slowing of the rate of desensitization in a dose-dependent manner (Figure 4B). Similar slowing of desensitization by amiloride was also observed with pH-7 evoked currents (Figures 4C,D).

We also tested if amiloride affects the kinetics of SSD. To measure time to SSD, we stepped from pH 8 to a desensitizing pH (6.0–7.0) for varying times, followed by a pH 6 test application (Figure 4E). Time to SSD was pH-dependent (faster at lower pH) and was significantly prolonged in the presence of 1 mM amiloride at all pH values tested (Figure 4F).

**FIGURE 5:**
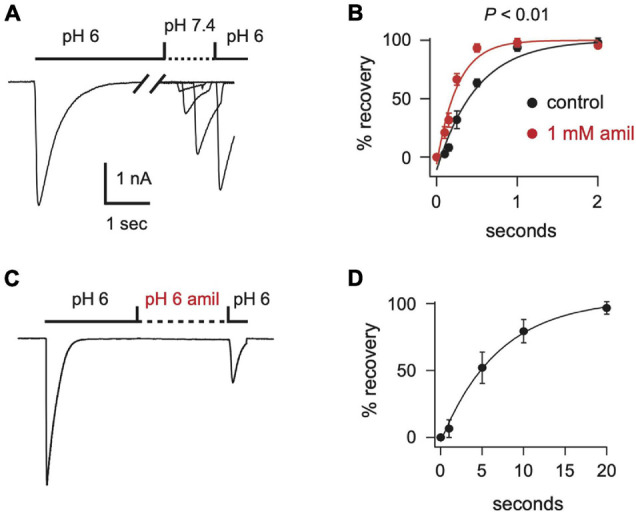
Amiloride increases the rate of recovery from desensitization of ASIC3. **(A)** Overlay of current traces showing recovery from desensitization. Current was desensitized with a 7 s application of pH 6 (only the first 2.5 s is shown). Cells were then exposed to pH 7.4 solution (containing 0 or 1 mM amiloride) for the indicated times (see *x* axis in **B**) before they were stimulated again with pH 6. Recovery is the percentage of current evoked by the second pH 6 application compared with the first. **(B)** Mean recovery data as collected with either no amiloride (control) or 1 mM amiloride in the pH 7.4 solution (*n* = 3–6 for each point). Lines are fits of single exponentials of the means. Students *t*-test of the τ’s calculated from the fits of the individual cells reveals that amiloride increased the rate of recovery (*p* < 0.01; *n* = 6). **(C)** Current evoked by a 10 s application of pH 6 without amiloride, followed by an exposure to a pH 6 solution containing 1 mM amiloride for variable times (see *x* axis in **D**), followed by a second pH 6 application without amiloride. After a 20 s exposure to the pH 6 solution with amiloride the ratio of the current evoked by the second pH 6 application compared to the first pH 6 application was 0.23 ± 0.04 (*n* = 8). **(D)** Mean normalized recovery data as collected in **(C)**. Data was normalized to the percentage of recovery at 20 s (*n* = 3–6). Line is fit of single exponential of the means (τ = 6.98 s).

After acid-evoked desensitization, ASIC channels require exposure to a more alkaline pH for some time before they can again be activated by acidic solution. We found that amiloride significantly increased the rate of recovery from desensitization of ASIC3 currents (Figures 5A,B). Moreover, amiloride caused ASIC3 channels to recover from desensitization even in the absence of exposure to an alkaline pH. The addition of amiloride at pH 6 to a completely desensitized pH 6-evoked current caused 23% recovery from desensitization within 20 s (Figures 5C,D).

### Extracellular Ca^2+^ Is Required for Amiloride Potentiation, While Inhibiting the Effects of Amiloride on Steady-State Desensitization

**FIGURE 6:**
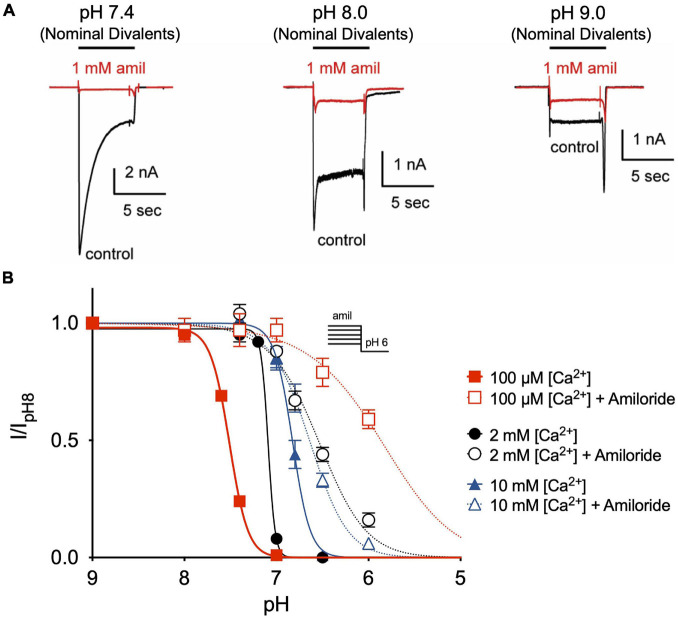
Amiloride potentiation requires divalent cations. **(A)** Overlay of representative ASIC3 currents evoked by stepping from pH 8 (containing 2 mM Ca^2+^ and 1 mM Mg^2+^ and no amiloride) to solutions without added Ca^2+^ and Mg^2+^ (nominal divalents) at pH 7.4, 8.0, and 9.0 in the absence (control) or presence of 1 mM amiloride. **(B)** pH dose-response curves for steady-state desensitization (SSD) recorded in either 100 μM, 2 mM, or 10 mM extracellular Ca^2+^ (no added Mg^2+^) in the desensitizing solutions in the absence of amiloride (pH_50_ = 7.51, 7.09, and 6.82, respectively) and in the presence of 1 mM amiloride (pH_50_ = 5.86, 6.57, and 6.64, respectively; *n* = 3–8 for each data point).

Divalent cations such as Ca^2+^ have previously been shown to stabilize ASIC channels in the closed state and that removal of extracellular Ca^2+^ opens ASIC3 channels (Immke and McCleskey, 2001; Babini et al., 2002; Immke and McCleskey, 2003). Similarly, we found that extracellular solutions without added Ca^2+^ or Mg^2+^ (nominal divalents) generated large ASIC3 currents at neutral and even alkaline pH solutions (Figure 6A). However, whereas amiloride potentiated currents evoked at pH 7.4 and 8.0 in the standard extracellular solution containing 2 mM Ca^2+^ and 1 mM Mg^2+^ (Figures 2C–F), amiloride inhibited currents in the absence of added divalents (Figure 6A), implying that extracellular divalent cations are required for amiloride potentiation.

Next, we tested if extracellular Ca^2+^ alters the effect of amiloride to inhibit ASIC3 pH-dependent SSD. By reducing extracellular Ca^2+^ to 100 μM, we found a significant shift to the left in the pH-dependence of SSD (Figure 6B). However, the addition of 1 mM amiloride to the 100 μM Ca^2+^ solution caused an even greater shift to the right than in the presence of 2 mM Ca^2+^. Conversely, increasing extracellular Ca^2+^ to 10 mM caused a shift to the right in SSD compared to that in 2 mM Ca^2+^, and the addition of 1 mM amiloride produced only a slight further shift to the right. Thus, these data indicate that extracellular Ca^2+^ inhibits, and possibly competes, with the effect of amiloride to inhibit SSD of ASIC3.

### Amiloride Effects Are Voltage-Dependent and Others Are Voltage-Independent

**FIGURE 7:**
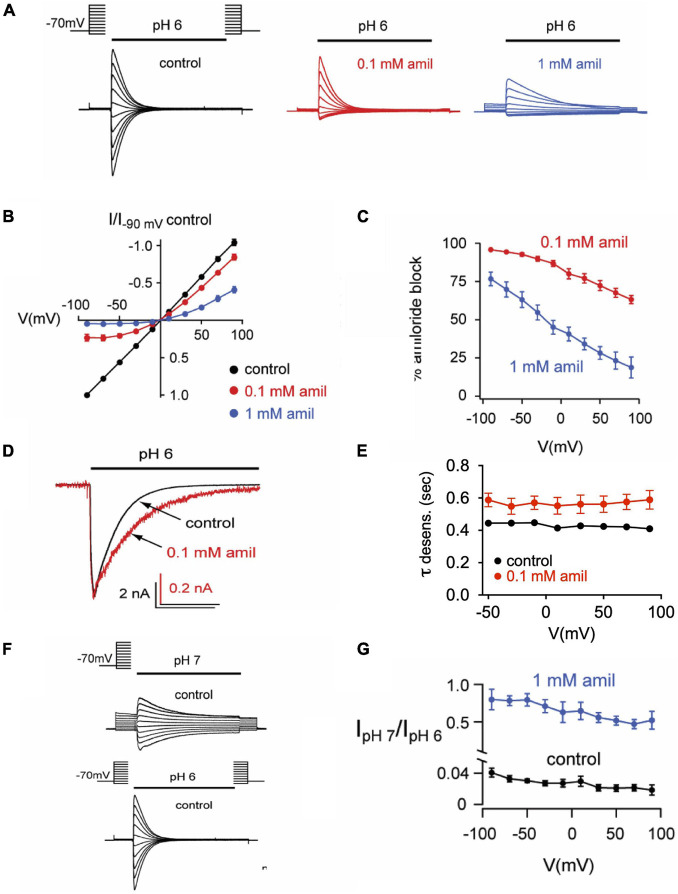
Effects of amiloride on ASIC3 are voltage-dependent and voltage-independent. **(A)** Overlays of pH 6-evoked ASIC3 currents recorded during steps to various membrane potentials (−90 to + 90 mV). The test solution (pH 6) contained 0 (control), 0.1, or 1 mM amiloride. The intracellular (pipette) and extracellular solutions contained symmetrical NaCl concentrations. **(B)** Current vs. voltage curves for the data in *A*. Data are normalized to the currents evoked at -90 mV in no amiloride (*n* = 17) vs. 0.1 mM (*n* = 7) and 1 mM (*n* = 10) amiloride. **(C)** Mean percentage of pH 6-evoked current block by 0.1 mM (*n* = 10) and 1 mM (*n* = 6) amiloride at the indicated voltages. **(D)** Overlays of pH 6-evoked normalized currents in the absence (control) or presence of 0.1 mM amiloride. **(E)** Mean time constants of desensitization (τ) recorded in 0 (*n* = 21) or 0.1 mM *(n* = 7) amiloride at the indicated voltages. **(F)** Overlays of pH 7- and pH 6-evoked currents recorded during steps to various membrane potentials (−90 to + *90 mV*). **(G)** Mean ratios of pH 7- to pH 6-evoked current amplitudes recorded in test solutions containing 0 mM (*n* = 3) or 1 mM (*n* = 3) amiloride at the indicated voltages.

Previous research on mutant ASIC2 channels showed that the blocking effects of amiloride were voltage-dependent, likely due to the influence of the membrane electrical field on the positively charged amiloride within the pore (Adams et al., 1999). We further tested the voltage-dependence of amiloride blocking and modulatory effects on ASIC3 (Figure 7). On pH 6-evoked currents, amiloride has a significant blocking effect at negative membrane potentials that lessens with increasing positive membrane potentials (Figures 7A–C). Although the blocking effect of amiloride was voltage-dependent, the prolongation of desensitization of ASIC3 by amiloride was not affected by voltage (Figures 7D,E). To assess the voltage-dependence of amiloride-mediated potentiation, the ratio of pH 7- to pH 6-evoked currents in the presence and absence of amiloride (1 mM) was examined to normalize against the blocking effects of amiloride (Figures 7F,G). Amiloride increased the pH 7- to pH 6-evoked current ratio equally across the voltage-gradient by approximately twenty-fold. This implies that the potentiating effects of amiloride to increase pH 7-evoked current is independent of voltage.

### The Non-proton Ligand Sensing Domain Is Required for Shifts in Steady-State Desensitization by Amiloride

**FIGURE 8:**
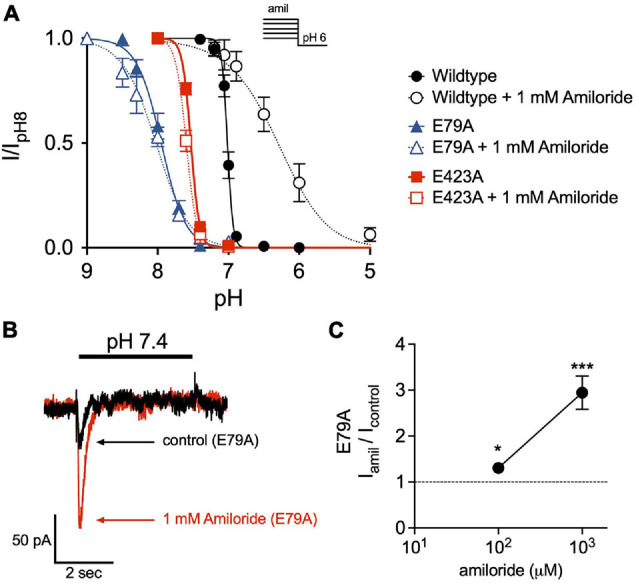
The non-proton ligand-sensing domain is required for the amiloride-induced acidic shift in steady-state desensitization. **(A)** pH dose-response curves for steady-state desensitization (SSD) in ASIC3 wildtype, E79A, and E423A under control conditions (no amiloride) and with the 1 mM amiloride concentration solution normalized to the currents evoked by stepping from pH 8 to pH 6 (*n* = 4–8 for each point). Lines are fits of the Hill equation (pH_50_ = 7.01, 7.95, and 7.53 under control conditions for wildtype, E79A, E423A, respectively; with 1 mM amiloride pH_50_ = 6.31, 8.02, and 7.59) **(B)** Representative superimposed E79A pH 7.4-evoked currents in the absence and presence of 1 mM amiloride. **(C)** Dose-response effect of amiloride 0.1 and 1 mM on pH 7.4-evoked currents in ASIC3 E79A (*n* = 8; *p = 0.02 0.1 mM vs. control; ***p < 0.001 1 mM vs. control, one-way ANOVA).

Disruption of a purported non-proton ligand sensing domain (E79 and E423) was previously shown to significantly blunt the stimulatory effect of amiloride on ASIC3 currents (Li et al., 2011). Similarly, we found that both E79A and E423A ASIC3 mutants shifted the pH-dependence of SSD toward more alkaline pH (E79A pH_50_ = 7.95, E423A pH_50_ = 7.53) when compared to wildtype (pH_50_ = 7.01), and completely mitigated the effect of amiloride on SSD (Figure 8A). Both E79A and E423A ASIC3 mutants displayed the same degree of potency of amiloride block of pH 6-evoked currents (data not shown). On the other hand, E79A did not fully abolish potentiation by amiloride. Due to a significant alkaline shift in the activation curve (Alijevic and Kellenberger, 2012), E79A channels generated small transient currents at pH 7.4, and current amplitude was increased in the presence of 1 mM amiloride (Figures 8B,C).

### Disruption of the Amiloride Pore Binding Site Diminishes Potentiating Effects

**FIGURE 9:**
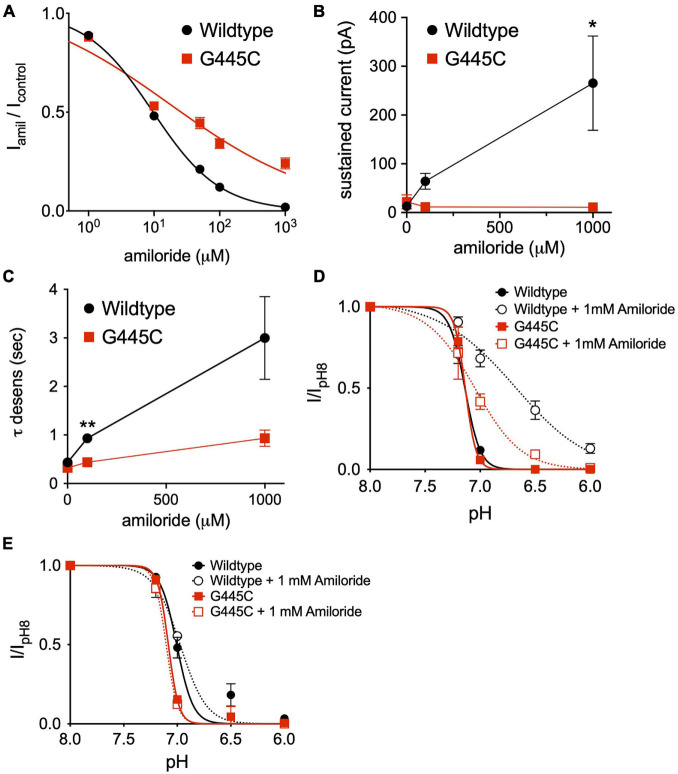
An amiloride binding site within the pore of ASIC3 contributes to paradoxical block and potentiation. **(A)** Dose-response curves for amiloride inhibition of pH 6-evoked currents on ASIC3 wildtype and G445C mutant channels. Lines are fits of Hill equation (IC_50_ = 9.47 μM (wildtype, *n* = 10) and 17.3 μM (G445C, *n* = 9; *p* < 0.001 two-way ANOVA) **(B)** Mean pH 6.8-evoked sustained current amplitudes measured from ASIC3 wildtype (*n* = 14) or G445C mutant channels (*n* = 5) in 0, 0.1, 1 mM amiloride (*p* = 0.13 two-way ANOVA with multiple comparisons: *p = 0.03 wildtype vs. G445C at 1 mM amiloride). **(C)** Mean time constants of desensitization (τ) of pH 6.8-evoked currents measured from ASIC3 wildtype (*n* = 18) or G445C mutant channels (*n* = 14) in 0, 100, 1,000 μM amiloride (*p* < 0.001 two-way ANOVA with Bonferroni *post hoc* showing **p < 0.001 for wildtype vs. G445C at 100 **(D)** pH dose-response curves for steady-state desensitization (SSD) in ASIC3 wildtype (*n* = 10) or G445C mutant channels (*n* = 5–12) recorded at -70 mV holding potential. Lines are fits of the Hill equation [pH_50_ = 7.13 (wildtype), 6.70 (wildtype + 1 mM Amiloride), 7.13 (G445C), 7.05 (G445C + 1 mM Amiloride)] **(E)** pH dose-response curves for SSD in ASIC3 wildtype (*n* = 10) or G445C mutant channels (*n* = 2) recorded at + 50 mV holding potential. Lines are fits of the Hill equation [pH_50_ = 7.08 (wildtype), 7.00 (wildtype + 1 mM Amiloride), 7.10 (G445C), 6.97 (G445C + 1 mM Amiloride)].

The capacity to fully characterize the potentiating effect of amiloride on ASIC3 is hindered by its simultaneous inhibition of the channel. Thus, we hypothesized that mutation of a purported amiloride blocking site within the second transmembrane-spanning domain that sits just above the pore (G445) would unmask the full potentiating effect of amiloride on ASIC3 (Schild et al., 1997; Alijevic and Kellenberger, 2012). Expressed alone, G445C generated only small currents requiring very acidic pH for activation (pH < 5.0). Therefore, we co-expressed G445C with wildtype ASIC3 at a 10:1 ratio, respectively, and found that the resultant pH-evoked currents had properties different than those generated when either G445C or wildtype ASIC3 were expressed alone, indicating the formation of heteromultimeric channels. As anticipated, G445C mutation partially blunted the inhibitory action of amiloride on ASIC3 currents when compared to wildtype ASIC3 channels (G445C IC_50_ = 17.3 μM vs. Wildtype IC_50_ = 9.47 μM, Figure 9A). Unexpectedly, G445C also blunted the potentiating effects of amiloride on sustained current observed at pH 6.8 (Figure 9B). Furthermore, G445C abrogated the effects of amiloride to slow the rate of desensitization (Figure 9C), and the shift in pH-dependent SSD (Figure 9D). To further investigate the role of the pore binding site in mediating the effects of amiloride, SSD measurements were carried at a positive voltage (+50 mV) to generate outward pH-evoked currents. Interestingly, under positive voltage, the effect of amiloride on SSD was abrogated in both wildtype and G445C channels (Figure 9E), thereby suggesting that the shift in SSD requires an amiloride binding site within the pore that is exposed to changes in the membrane electrical field. Together, these data demonstrate that the purported amiloride binding site within the pore contributes to both inhibition and potentiation by amiloride.

## Discussion

Amiloride is the classic small-molecule blocker of ASICs. It has been used to probe the structure-function and gating mechanisms of ASICs and has served as a *in vivo* tool to explore the physiological role of ASICs. And yet, recent studies have shown complex actions of amiloride on ASICs—including the capacity to potentiate pH-evoked current—which complicates our understanding of amiloride as a pure channel blocker. Here, we further explored the actions of amiloride on one of the ASIC isoforms, ASIC3. We confirmed that amiloride can both inhibit and potentiate ASIC3 pH-evoked current, dependent upon both the concentration of amiloride and the pH of activating solution. Much of the potentiating capacity of amiloride occurs *via* an alkaline shift in the pH dose-response of activation, and an acidic shift in the dose-response of SSD, which generates larger sustained “window” currents at the “foot” of the activation pH range. The variable effects of amiloride on ASIC3 occurred at varying potencies, varying voltage-dependency, and mutation of different sites within the channel variably disrupted the effects of amiloride—all suggesting that amiloride exerts its effects through multiple domains within the channel, and perhaps binds to multiple different sites. In particular, disruption of the purported blocking site within the pore not only abrogated inhibition, but also diminished the potentiating effects of amiloride. Moreover, we found the acidic shift of SSD induced by amiloride was voltage-dependent, suggesting a site with the transmembrane region is required for the potentiating effects of amiloride.

### Amiloride Binds to Acid-Sensing Ion Channel 3 in Multiple Different Conformational States and Modulates Several Different Gating Transitions

**FIGURE 10:**
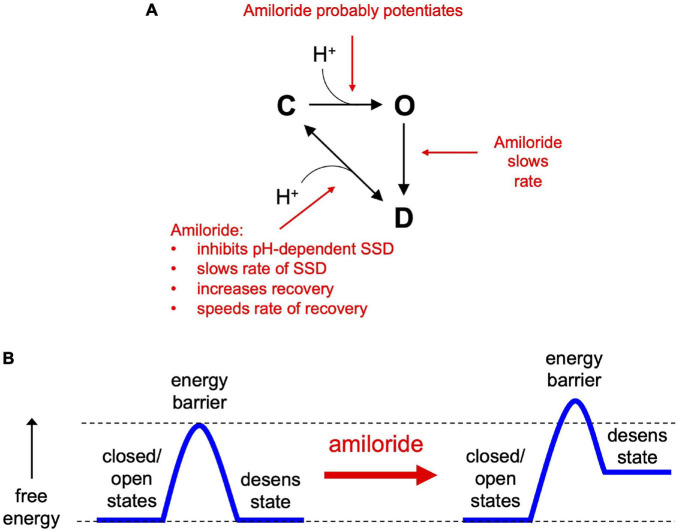
The effects of amiloride on ASIC3 gating properties. **(A)** A simple state model highlighting the various effects of amiloride on ASIC3 gating [closed (C), open (O), and desensitized (D) states]. **(B)** An energy barrier model demonstrating how amiloride increases the barrier to the desensitized state and increases the free energy of the desensitized state.

In the simplest of gating models, ASICs transition from a closed to an open state upon exposure to H^+^ (Figure 10A). From the open state, channels rapidly transition into a closed desensitized state depending on the pH of the activation solution. At less acidic non-activating H^+^ concentrations, channels may also transition directly from a closed to a desensitized state, as measured with SSD protocols (Figure 3). While this scheme in Figure 10A does not account for multiple closed, open, and desensitized states, it serves our purpose to highlight the multiple effects of amiloride on ASIC3 gating. Much of the potentiating effects of amiloride on ASIC3 can be described by a destabilization of the desensitized state (Figures 10A,B). Amiloride inhibited the pH-dependence of SSD (Figure 3), slowed the rate of SSD (Figures 4E,F), and slowed the rate of desensitization from the open state (Figures 4A–D). Previous work did not find an effect of amiloride on the rate of ASIC3 open-channel desensitization (Besson et al., 2017)—the reason for the discrepancy could be due to different channel constructs or different cell expression systems. The effect of amiloride to destabilize the desensitized state is perhaps best illustrated by amiloride’s effect on recovery from desensitization. Typically, this process requires exposure to an alkaline pH (presumably to remove H^+^ bound to the channel), however recovery occurred in 1 mM amiloride even when pH remained at 6.0 (Figures 5C,D). Additionally, amiloride increased the rate of recovery from desensitization (Figure 5B).

The slowing of desensitization from either the open or closed states implies that amiloride causes an increase in the energy barrier to transition to the desensitized state (Figure 10B). The inhibition of pH-dependent SSD, and the potentiation of recovery from desensitization, imply that amiloride also generates an increase in the free energy (destabilization) of the desensitized state relative to the open and closed states.

As previously reported, amiloride also probably potentiates the pH-dependent activation of ASIC3 (Besson et al., 2017). We found that amiloride generated currents at pH solutions of pH 7.4 and 8.0 (ranges that do not generate ASIC3 current in the absence of amiloride), suggesting that amiloride caused an alkaline shift in pH activation. However, it should be noted that the pH dose-response of activation in the presence of amiloride cannot be accurately assessed due to: (1) amiloride inhibition of currents activated by more acidic pH solutions leads to an overestimation of pH sensitivity, and (2) depending on the rates of desensitization and activation, an alkaline shift in SSD could alone account for a shift in the foot of the pH-dose response of activation (Grunder and Pusch, 2015).

Amiloride likely binds to ASIC3 in all conformational states. Inhibition of SSD and slowing the rate of open-channel desensitization implies that amiloride binds to both the closed and open states, respectively. Amiloride can also bind to the desensitized state since it facilitates recovery from desensitization. However, which sites within the channel that bind amiloride to generate these various functional effects is less clear.

### Amiloride Competes With Extracellular Ca^2+^ to Modulate Acid-Sensing Ion Channel 3

Among ASIC isoforms, ASIC3 has the unique capacity to be activated at neutral pH by diminished extracellular Ca^2+^ (Immke and McCleskey, 2003; Paukert et al., 2004; Zhang et al., 2006). Changes in extracellular Ca^2+^ also cause significant shifts in pH-dependent activation and recovery from desensitization (Immke and McCleskey, 2001, 2003). Here we found that in the absence of extracellular Ca^2+^, amiloride did not potentiate ASIC3 and only blocked current. Additionally, changes in extracellular Ca^2+^ also generated large shifts in the pH dose-response of SSD, demonstrating that Ca^2+^, like amiloride, inhibits pH-dependent SSD. Moreover, we show that amiloride and Ca^2+^ compete to inhibit SSD, suggesting that they do so through a common mechanism, and perhaps a common binding site. Interestingly, Ca^2+^ also competes with the neuropeptide FMRFamide to inhibit pH-dependent SSD of ASIC1b/3 heteromeric channels (Chen et al., 2006). The Gründer group has characterized the blocking and modulating effects of Ca^2+^ on ASIC1a and concluded that these varying effects occur *via* different Ca^2+^ binding sites in the channels that have different binding affinities—the blocking effect occurs with high-affinity (low micromolar range) (Immke and McCleskey, 2003), and the modulating effect occurs *via* a low-affinity site (∼2 mM) (Babini et al., 2002). They found that Ca^2+^ competes for amiloride block of ASIC1a, and this competition was abolished with mutation of a purported Ca^2+^ binding site within the outer entrance of the pore (Paukert et al., 2004). Work to identify the Ca^2+^ modulating domains within ASICs have identified several different sites, dependent on the isoforms studied (Babini et al., 2002; Sherwood et al., 2009; Zuo et al., 2018). Further complicating the competitive interaction of Ca^2+^ with amiloride is that the presumed shared binding site(s) are undoubtedly protonated at lower pH (Immke and McCleskey, 2003; Paukert et al., 2004). Thus, we postulate that the myriad of modulatory and blocking effects of amiloride are the result of a complex competitive interaction between amiloride, Ca^2+^, and H^+^ at probably more than one site in the channel. In support of this idea, amiloride causes both the pH- activation and SSD dose-response curves to be less steep (Figure 3C; Besson et al., 2017), implying that amiloride disrupted the observed cooperativity of H^+^-binding to the channel (Bonifacio et al., 2014). A competition between shared binding sites could very well explain the variable effects of amiloride to either block or potentiate, depending on both amiloride and H^+^ concentrations.

### Amiloride Likely Binds to Multiple Sites in Acid-Sensing Ion Channels and Modulates *via* Different Mechanisms

Previous work has demonstrated that amiloride has the capacity to bind to multiple sites within DEG/ENaC channels, including ASICs. The blocking effects of amiloride have been principally explored in ENaC, leading to identification of a purported binding site within the outer vestibule of the pore (McNicholas and Canessa, 1997), including a critical conserved Glycine residue (G445 in rat ASIC3) (Schild et al., 1997), although other sites may also contribute to ENaC block (Ismailov et al., 1997). However, amiloride is also known to inhibit other proteins including other sodium channels and Na^+^/H^+^ exchangers (Kellenberger and Schild, 2002), indicative of the non-specific binding properties of amiloride. Both molecular modeling of amiloride docking within human ASIC1 and crystallization of chicken ASIC1 in the presence of amiloride have demonstrated that amiloride has the potential to bind to multiple different sites within ASIC channels (Qadri et al., 2010; Baconguis et al., 2014).

Importantly, it should be noted that binding cannot necessarily be inferred from observed functional effects on the channel (Colquhoun, 1998, 2006). Ligand-induced changes in channel activity require binding of the ligand and then subsequent steps that typically involve conformational (gating) changes. Amiloride could bind to a particular site, and then other domains are required for the subsequent conformational changes to a different state. For example, we confirmed that mutation of a purported non-proton ligand sensor (E79 and E423) largely abolished the effect of amiloride to inhibit pH-dependent SSD (Li et al., 2011; Besson et al., 2017). While amiloride could very well bind to this site (Yu et al., 2011), it is also possible that amiloride binds to a different site and that E79 and E423 are required for the subsequent conformation changes to the desensitized state, as these sites have proven to be critical for ASIC3 desensitization (Cushman et al., 2007). In fact, we found that mutation of E79 did not abolish amiloride potentiation of pH 7.4-evoked currents, suggesting that this site does not underlie the alkaline shift in the pH activation curve caused by amiloride. This is similar to that described with GMQ, whereby mutation of this site abolishes the amiloride shift of SSD, but not activation (Alijevic and Kellenberger, 2012). Thus, we conclude that the purported non-proton ligand sensing site does not account for all of the potentiating effects of amiloride or GMQ on ASIC channels.

On the other hand, our studies on the voltage-dependence of the various amiloride blocking and modulating effects lends direct insight as to where amiloride binds to produce these effects. Similar to studies in a mutant ASIC2a channel (Adams et al., 1999), we found that the blocking effect of amiloride on ASIC3 was voltage-dependent; holding the cell at positive voltages caused relief of amiloride block, presumably by electrostatically driving the positively charged amiloride from a binding site within the membrane-spanning domains. Conversely, the potentiation of pH 7-evoked current amplitude and the slowing of open-state desensitization were both voltage independent, implying that the amiloride binding site inducing these modulatory effects is outside of the membrane electrostatic field. Surprisingly, we also found amiloride inhibition of pH-dependent SSD was voltage-dependent; a finding supported by our results that mutation of a purported amiloride binding site on the second transmembrane-spanning domain (G445) largely mitigated the effect of amiloride on SSD. These results suggest that a binding site just above the pore contributes to both channel block and potentiation of pH-evoked currents. Such paradoxical effects could be explained by a “foot-in-the-door” phenomenon (Armstrong, 1971), whereby amiloride binds deeply near the entrance of the pore to block ion permeation and at the same time obstructs a conformational change to the desensitized state. It is interesting that amiloride potentiation of pH-7 evoked current amplitude was voltage-independent whereas its effect on SSD was voltage-dependent, suggesting that these modulatory effects occur through different amiloride binding sites. It is possible that the effects on SSD are mediated by an amiloride binding site within the transmembrane-spanning domains whereas the site(s) that leads to a shift in pH-dependent activation lies within the extracellular domains. It is also quite conceivable that amiloride binding to more than one site is required for a particular modulatory effect.

While much of our understanding of ASIC gating has focused on the extracellular domain sites where H^+^ and other ligands bind and subsequently impart conformational changes that are transmitted to the transmembrane domains, it is interesting that mutations within the transmembrane domains can also have profound effects on pH-dependent activation and desensitization. Besson et al. (2017) found that replacing the transmembrane domains of ASIC3 with those of ASIC1a generated a channel that no longer generated window currents at the foot of the pH-activation dose response and in the presence of amiloride the window currents were ∼20 times diminished in amplitude compared to the effect of amiloride on wildtype ASIC3. Thus, there appears to be a complex interplay between the functional effects of ligand-binding sites within the extracellular domain and transmembrane domains that is not well understood.

## Conclusion

The pharmaceutical industry is aware of the potential therapeutic benefits of ASIC modulation and has sought to engineer amiloride derivatives that have varying effects on channel function (Kuduk et al., 2009; Baron and Lingueglia, 2015). A better understanding of the modulatory mechanisms of amiloride on ASICs is critical to advancing this endeavor. Additionally, further insights into the modulation of ASICs by amiloride and other small-molecule derivatives will provide more context within studies utilizing these compounds to investigate the physiological role of ASICs *in vivo*. Here we report that amiloride can both block and modulate ASIC3 in a variety of ways and probably does so through multiple different binding sites. The mechanism underlying the modulatory effects of amiloride on ASICs is likely more complex than previously thought.

## Data Availability Statement

The raw data supporting the conclusions of this article will be made available by the authors, without undue reservation.

## Author Contributions

DM, NH, PS, and CB conceived and designed the studies. DM, NH, MG, DG, NK, AH, VS, and CB conducted the experiments and analyzed the data. DM, NH, and CB wrote the manuscript. All authors contributed to the article and approved the submitted version.

## Conflict of Interest

The authors declare that the research was conducted in the absence of any commercial or financial relationships that could be construed as a potential conflict of interest.

## Publisher’s Note

All claims expressed in this article are solely those of the authors and do not necessarily represent those of their affiliated organizations, or those of the publisher, the editors and the reviewers. Any product that may be evaluated in this article, or claim that may be made by its manufacturer, is not guaranteed or endorsed by the publisher.
